# Digital interventions for weight control to prevent obesity in adolescents: a systematic review

**DOI:** 10.3389/fpubh.2025.1584595

**Published:** 2025-05-23

**Authors:** Vincenzo De Luca, Michele Virgolesi, Claudia Vetrani, Sara Aprano, Federica Cantelli, Annamaria Di Martino, Lorenzo Mercurio, Guido Iaccarino, Francesco Isgrò, Pasquale Arpaia, Annamaria Colao, Maddalena Illario

**Affiliations:** ^1^Department of Public Health, Federico II University of Naples, Naples, Italy; ^2^Department of Wellbeing, Nutrition and Sport, Pegaso Telematic University, Naples, Italy; ^3^Italian Centre for the Care and Wellbeing of Patients with Obesity, Federico II University Hospital, Naples, Italy; ^4^Interdepartmental Research Centre on Healthcare Management and Innovation, Federico II University of Naples, Naples, Italy; ^5^Department of Clinical Medicine and Surgery, Federico II University of Naples, Naples, Italy; ^6^Department of Electrical Engineering and Information Technologies, Federico II University of Naples, Naples, Italy

**Keywords:** digital health, obesity prevention, adolescents, mHealth, nutrition

## Abstract

**Background:**

Obesity is a common, serious, and costly chronic disease of adults and children that poses serious long-term health risks. Recent global estimates from the World Health Organization (WHO) show that the number of adolescents living with overweight or obesity is now increasing in low- and middle-income countries, particularly in urban settings. Health interventions using information technology (IT), especially diet and activity tracking, can lead to significant reductions in weight status.

**Objective:**

This systematic review aimed to map IT-supported interventions designed to prevent obesity in adolescents, promoting healthy nutrition and physical activity. Methods: The Preferred Reporting Items for Systematic Reviews and Meta-Analyses checklist was used. A search of the electronic databases Medical Literature Analysis and Retrieval System Online (MEDLINE) and Excerpta Medica Database (EMBASE) was conducted using search terms in various combinations appropriate to the research objective. The Effective Public Health Practice Project (EPHPP) quality assessment tool was used to assess quality.

**Results:**

A total of 21 English language studies were eligible for inclusion. The systematic review synthesized information on weight control IT-supported intervention trials to prevent obesity, their domains of intervention, implementation setting, digital tool adopted, and the outcomes assessed.

**Conclusion:**

The interventions included in the present study mainly concern nutritional aspects and physical activity, but motivational and psychological support also play a fundamental role in their success.

**Systematic review registration:**

https://www.crd.york.ac.uk/PROSPERO/view/CRD42024412913, identifier [CRD42024412913].

## 1 Introduction

Obesity is a common, serious, and costly chronic disease of adults and children that poses serious long-term health risks. According to the World Health Organisation (WHO) global estimates, in 2019, 38.2 million children under the age of 5 years were overweight or obese. This number is now on the rise in low- and middle-income countries, particularly in urban settings. In 2016, over 340 million children and adolescents aged 5–19 were overweight or obese. The rise has occurred similarly among boys and girls: in 2016, 18% of girls and 19% of boys were overweight (1). In Europe in 2007, the WHO Regional Office established the European Childhood Obesity Surveillance Initiative (COSI) in response to the need for standardized surveillance data on the prevalence of children living with overweight or obesity among school-aged children. The latest COSI data collection was carried out in primary schools in 33 countries in 2018–20, including children aged 6–9 who had their weight and height measured. The prevalence of children aged 7–9 living with overweight or obesity is 29%, with a higher value among boys (31%) than girls (28%). However, the data show considerable variability across countries, with an overall prevalence of overweight ranging from 43% in Cyprus to 6% in Tajikistan, and an overall prevalence of obesity from 19% in Cyprus to 1% in Tajikistan (2). Italy is one of the countries with the highest prevalence: 39% overweight (including obesity) and 17% obesity among boys and girls aged 8 years old (2). In 2019, 206 schools (96.3%) in Campania (a region in southern Italy with about 6 million inhabitants) participated in the “Okkio alla Salute” survey for the COSI initiative, with 3,453 children enrolled. The survey suggests that 6.2% (*n* = 214) of children were in conditions of severe obesity, 12.6% (*n* = 435) were obese, and 25.4% (*n* = 877) were overweight. Regarding the perception of parents, as many as 63.3% of mothers of overweight children and 14.5% of mothers of obese children believe that their child is of normal weight or underweight (3). The “WHO Global Strategy on Diet, Physical Activity and Health” describes the importance of increasing general awareness and understanding of the effects of diet and physical activity on health, and this approach encourages health systems to collaborate with civil society, the private sector and the media to achieve this important goal (4). In 2023, the European Commission launched the “Joint Action for the implementation of best practices and research results on Healthy Lifestyle for the health promotion and prevention of non-communicable diseases and risk factors” (JA-Health4EUkids) to achieve results in terms of promoting healthy lifestyles and preventing childhood and school-age obesity. This action includes increasing physical activity and promoting healthy nutrition in boys and girls from the first 1.000 days of life, in families and communities. The JA-Health4EUkids aims to generate more interest and engagement in tackling childhood obesity in EU Member States, developing and applying a Health in All Policy (HiAP) approach (5). Given the multifactorial nature of obesity and its variability in terms of severity and implications for health, treating childhood obesity must be integrated at the healthcare level and involve professionals (6). The primary intervention for weight control is universal, targeting the entire population to reduce the incidence of obesity. The secondary intervention, defined as selective prevention, is aimed at the groups at risk, i.e., children of parents, children with low birth weight due to intrauterine growth restriction, and macrosomic children who tend to anticipate the adiposity rebound (7). Recent literature points out that parental body shape and an “obesogenic environment,” including increasingly early exposure to technological devices, are risk factors of early rebound adiposity (8, 9). Selective prevention is aimed at reducing the incidence of obesity in those already experiencing obesity from worsening the severity of their overweight and/or developing complications. It affects the prodromal risk factors of cardiovascular and metabolic mortality and is therefore aimed at preventing avoidable mortality. The tertiary prevention is aimed at children and adolescents affected by overweight or obesity, along with associated comorbidities, and is aimed at reducing complications (10). Several studies suggest that technology is a powerful ally in maintaining a healthy diet and lifestyle in the young population (11). In particular, digital interventions monitoring diet and physical activity can lead to significant reductions in weight status (12). Technological advances have made smartphone sensors more stable for real-time data collection, allowing them to be shared and processed for multiple analyses (13). Given the widespread use of smartphones in our daily lives—with 7.2 billion users worldwide, with peaks above 70% even in low- and middle-income countries—mHealth solutions can improve accessibility, quality, and efficiency. They also enhance the cost-effectiveness of interventions for the medium and long-term management of patients suffering from obesity or at potential risk group (14, 15). Information Technologies (IT) can contribute to the healthcare outcomes if they are adequately integrated into end users’ care processes, work routines and daily life. Technology can support the intervention of caregivers and health professionals (pediatricians, nutritionists, kinesiologists), providing more information on lifestyle behaviors and improving communication. Still, they cannot replace them in any way. Despite the potential of mHealth to support this aim, further adaptation to the context and a service delivery business model are needed. In Italy, I-Perseo project, financed by the National Center for Diseases Prevention and Monitoring (Centro Nazionale per la Prevenzione e il Controllo delle Malattie—CCM) of the Italian Ministry of Health, aims to address the unmet needs of overweight and obese adolescents, through the design and implementation of an innovative diagnostic and therapeutic pathway, integrating areas and levels of service delivery through digital solutions (16). There are many digital solutions, especially mHealth for interventions on obesity (17), but they lack evidence to assess clinical and organizational efficacy, technical usability, and patients’ engagement and satisfaction (18, 19). This review aims to identify interventions that investigated interventions for weight control to prevent obesity in adolescents, supported by digital solutions, identifying the target population, the setting and the level of intervention.

## 2 Materials and methods

### 2.1 Study design

The present systematic review was performed following the Preferred Items for Systematic Reviews and Meta-Analyses (PRISMA) guidelines (20), and the study protocol was registered with PROSPERO (Registration No CRD42024412913). In March 2023, articles on digital interventions to prevent obesity among adolescents were searched through the Medical Literature Analysis and Retrieval System Online (MEDLINE) and Excerpta Medica database (EMBASE) electronic databases. Clinical trials or randomized controlled trial articles published in peer-reviewed scientific journals between 2013 and 2023 (until February 28, 2023) were included in the search. The review used the PICOS framework reported in Table 1 to identify search terms. Only articles written in English have been included. The terms used for the search at the title and abstract level were reported in Table 2, combined with the Boolean operators “OR” and “AND.”

**Table 1:** PICOS framework to identify search terms.

Population	Adolescents of both sexes, aged between 12 and 17 years
Implementation	Digital solutions to monitor any type of health condition or to promote healthy eating, physical activity and weight control
Comparator	Any type of pre- and post-intervention comparison
Outcome	Results related to healthy lifestyles
Study	Observational studies and experimental

**Table 2:** Search terms used for the present review’s search strategy.

Key search terms	Related search terms
Nutrition and obesity	(obesity OR nutrition OR obese OR overweight OR malnutrition)
Adolescents	(young people OR adolescents OR juvenescent OR teenagers OR teens)
Digital intervention	(digital intervention OR digital health OR mHealth OR digital solution OR information technologies OR digital tool)
Nutritional intervention	(Nutritional intervention OR healthy nutrition OR obesity prevention OR health promotion OR primary nutritional intervention OR nutritional counseling OR food education)

### 2.2 Data extraction

Duplicate entries from the database search have been eliminated. Three reviewers subsequently independently reviewed the title and abstract for eligibility. All articles were read before determining which studies should be included. Each reviewer recorded their inclusion and exclusion decisions on an electronic form, and differences were discussed and resolved by consensus. The main discussions were about excluding studies that targeted specific patient groups, whose nutritional and weight control interventions aimed to treat the disease. Four reviewers performed data extraction and harmonization. The data were reported following a standardized approach based on the following elements: (1) general information (name of the author and year of publication of the study), (2) age of the sample, (3) size of the sample, (4) sex of the sample, (5) type of study, (6) context of implementation, (7) level of intervention, (8) purpose of the study, (9) procedures, (10) digital tool adopted, (11) inclusion criteria, (12) measures, and (13) results. The resolution of disagreements was carefully recorded. A copy of the “as extracted” data (in addition to the consent data) was recorded on an electronic form. Six items were used to assess the study quality. The above elements include study design, confounding variables, data collection methods/tools, whether raters and participants were “blinded,” reports of withdrawals and dropouts. A weak, moderate, or strong score was assigned for each item according to Thomas et al.’s standardized guide and dictionary (21). The rating of the six components determines the overall study rating. Those with no weak and at least four strong ratings are considered strong. Those with less than four strong ratings and one weak rating are considered moderate. Finally, those with two or more weak ratings are considered weak. Three reviewers performed this process separately. Differences were discussed and resolved by consensus.

## 3 Results

### 3.1 Study selection

**Figure 1:**
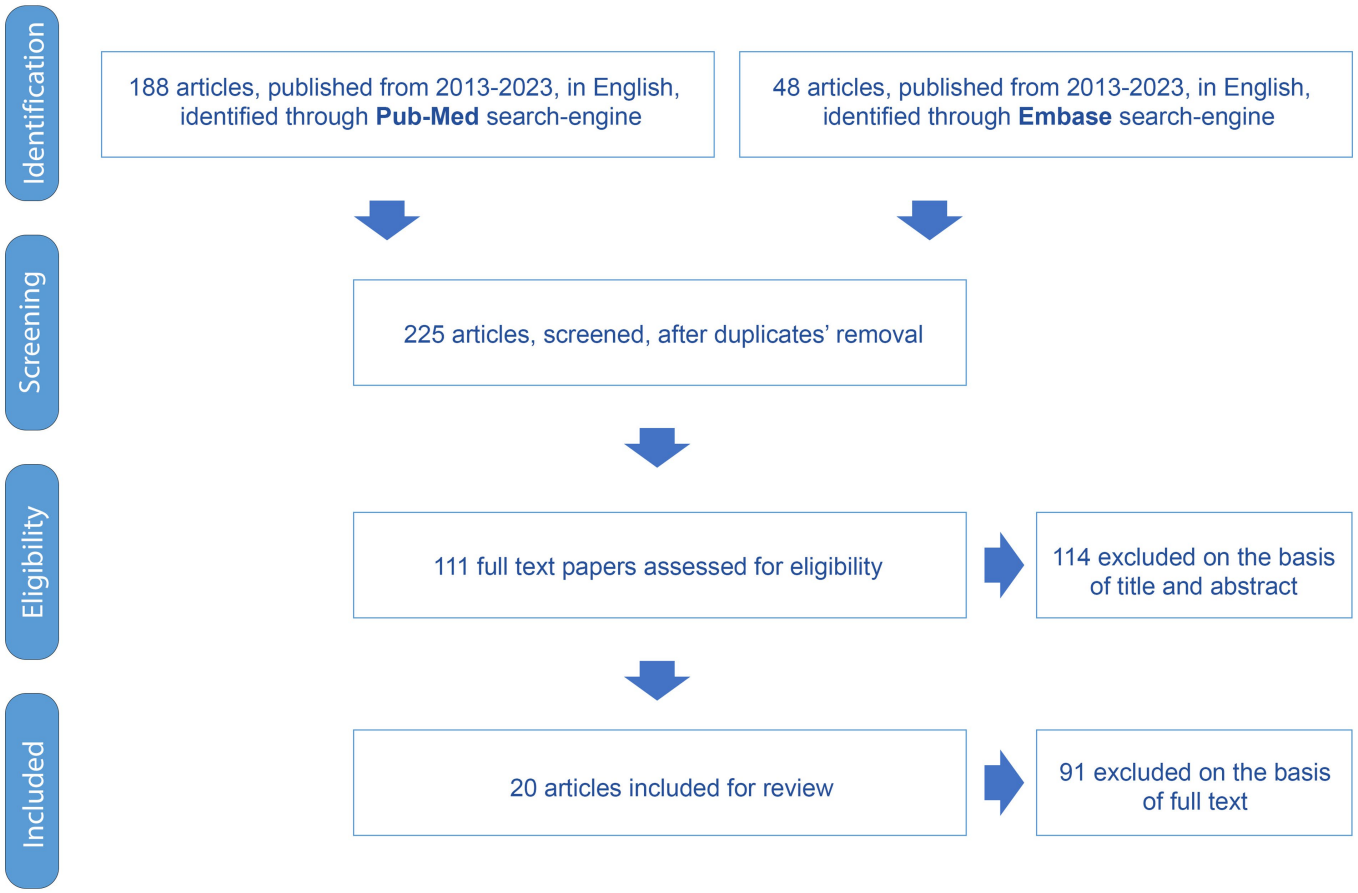
Flowchart of the PRISMA study selection procedure.

As shown in Figure 1, 236 articles were identified by searching for the selected electronic databases. Two hundred twenty-five remained for eligibility after the removal of duplicates. One hundred and fourteen articles were eliminated during the title and abstract screening phase. One hundred eleven articles were fully assessed, and 20 were chosen as pertinent for inclusion.

### 3.2 Study characteristics

Table 3 reports the characteristics of each study included in this review.

**Table 3:** Main characteristics of the studies included in the present review.

General Info	Sample info			Study type	Setting	Intervention level	Study purpose	Procedures	Digital tool	Inclusion criteria	Measures	Results
	Age	Size	Sex									
Stasinaki et al. (24)	10–18	42	Both	RCT	Health center	Tertiary prevention	The study aimed to assess the effects of counseling via a mobile app to support adolescents with overweight or obesity in adopting a healthy lifestyle, compared to a multi-component behavior-changing program.	During 5.5 months, a group received daily conversational agent counseling via mobile app, combined with standardized counseling (4 on-site visits). Controls participated in behavior-changing interventions (7 on-site visits).	Mobile App	> P.90 with risk factors or co-morbidities or BMI > P.97	BMI, waist-to-height ratio, physical performance, blood pressure, heart rate and stress parameters	BMI-SDS decreased significantly in the Control group and did not decrease in the Mobile app group. Muscle mass, strength and agility improved significantly in both groups; only the mobile app group significantly reduced their body fat. Average daily mobile app usage rate was 71.5%. Cortisol serum levels decreased significantly after biofeedback but with no association between stress parameters and BM I-SDS.
Vidmar et al. (25)	12–18	18	Both	Observational study	Health center	Secondary prevention	The study aimed to assess adherence to healthy nutrition and BMI change via a mobile app intervention to help adolescents without significant obesity comorbidity omit problem foods, avoid snacking and reduce meal size.	Height and weight were assessed at in-person visits at baseline, 3, and 6 months (program completion). BMI was calculated as kilograms per meter squared, and BMI Z-score (zBMI) and excess per cent over the 95th percentile (%BMIp95) were determined utilizing the CDC growth charts. Efficacy of the app intervention was compared to age-matched EMPOWER youth who completed six or more clinic visits.	Mobile App with coaching Text message with motivational phrases from a mentor Phone meetings with the mentor	Positive Yale Food Addiction Scale for Children (YFAS-c), without significant comorbidity	BMI Z-score (zBMI) and excess per cent over the 95th percentile (%BMIp95)	App participants exhibited a significant decrease in zBMI and %BMIp95 over the 6 months (*p* < 0.001 and *p* = 0.001), which was comparable to the age-matched EMPOWER program completers (*p* = 0.31 and *p* = 0.06).
Verswijveren et al. (28)	≥13	75	Both	RCT	School	Primary prevention	The study aimed at increasing physical activity among inactive adolescents through combining a wearable activity tracker with digital resources delivered via a private Facebook group	12-week intervention, grounded in social cognitive theory and behavioral choice theory, aimed at increasing physical activity among inactive adolescents through combining a wearable activity tracker with digital resources delivered via a private Facebook group	Mobile app with interactive individual or weekly missions Wearable activity tracker Social Media	Adolescents not engaging in regular organized physical activity or sport outside of school, not meeting physical activity guidelines of ≥60 min of moderate to vigorous physical activity (MVPA) every day	A series of mixed linear models were used to estimate intervention effects on physical activity and sedentary behavior at follow-up and on potential mediators after intervention	Adolescents in the intervention group (*n* = 75) engaged in higher sedentary time and lower light intensity at 6-month follow-up compared to the wait-list controls (*n* = 84). There were no intervention effects for moderate-to-vigorous–intensity physical activity.
Sousa et al. (36)	12–18	94	Both	Non-RCT	School	Primary prevention	The study aimed to assess the effectiveness of an e-therapeutic platform intervention for weight control and healthy lifestyle promotion programs compared to the conventional treatment	24 weeks of intervention in 10 thematic modules, with an average duration of 2 weeks through the e-therapeutic platform (Next.Step), in addition to the standard treatment program.	Mobile app for gaming Social Media (discussion forums, chat, and personalized messages)	≥ 95	BMI, behavioral variables, AWCQ—Adherence to Weight Control Questionnaire, ALP—Adolescent Lifestyle Profile and IWQOL—Impact Weight on Quality of Life, Usability questionnaire	The e-Therapeutic program led to a significant increase in health responsibility, whereas inconclusive results were found regarding the program’s effectiveness compared to the standard multidisciplinary intervention. The lack of significant differences between groups may be due to the reduced rates of program adherence and the high dropout rate.
Hagman et al. (26)	4.1–17.4	107	Both	Pragmatic clinical trial	Health center	Secondary prevention	The study aimed to show the real-world effectiveness of a digital support system in combination with a clinical visit for pediatric obesity lifestyle treatment	Before treatment, the child met a pediatrician for a physical check-up; a dietitian and a physiotherapist, if needed. Then the family has been trained on how to use the IT system. In-person meetings were scheduled every 3 months.	Mobile App integrated with Bluetooth body scale	Diagnosis of obesity	Weight and BMI Z-score	After 1 year, the BMI Z-score outcome was better for both sexes and all age classes in the group treated with the digital support system.
Ridgers et al. (23)	13–15	275	Both	RCT	School	Primary prevention	The study aimed to assess the effects of a wearable activity tracker combined with digital behavior change resources on moderate and vigorous physical activity of adolescents attending schools in socio-economically disadvantaged areas.	12 weeks multicomponent intervention that combined a Fitbit Flex (and accompanying app), online resources on digital behavior change and weekly challenges delivered via Facebook. Data were collected at baseline, immediately post-intervention and 6 months post-intervention (follow-up).	Mobile App for Physical Activity tracking Social media	Age and self-reporting that they did not engage in regular physical activity/sport	Time spent in moder- ate- to vigorous-intensity physical activity, steps taken, physical activity intensities, estimated energy expenditure, and sleep duration.	No significant differences were observed between intervention and wait-list control adolescents’ device-assessed MVPA immediately post-intervention. 6 months post-intervention, adolescents in the intervention group engaged in 5 min less MVPA per day than those in the wait-list control group.
Salahshornezhad et al. (29)	9–12	62	Fem.	RCT	School	Secondary prevention	The study aimed to evaluate the effect of a multi-component program including smartphone nutrition education, physical activity, and cognitive behavioral therapy (CBT), in the management of obesity and overweight among elementary school girls, in comparison with a traditional education method.	10 weeks of intervention, including smartphone games with some information at the beginning about the causes and complications of obesity, food sources of fiber and vitamins, and banned foods; aerobic exercise for 45 min three times per week; eight CBT Sessions by a clinical psychologist.	Mobile App for Gaming	BMI > 85th percentile	Anthropometric measurements; biochemical and metabolic lab data; Metabolic Equivalent Test (MET) and Dutch Eating Behavior Questionnaire (DEBQ)	Results not available
Ptomey et al. (30)	13–18	19	Both	Observational study	Home	Primary prevention	The study aimed to determine the feasibility of collecting a 3-day image-assisted food records (IAR) and doubly labeled water (TDEEDLW) data in intellectual and developmental disabilities adolescents to obtain preliminary estimates of validity and reliability for energy intake estimated by IAR.	Adolescents with IDD completed a 14-day assessment of mean daily energy expenditure using DLW. Participants were asked to complete 3-day IARs twice during the 14-day period (1 week apart). To complete the IAR, participants, assisted by a parent (if needed), were asked to fill out a hard-copy food record over 3 consecutive days (2 weekdays/1 weekend day) and to take before and after digital images of all foods and beverages consumed using a tablet. To assess test–retest reliability of the IAR, participants were asked to complete the IAR for the same 3 days (e.g., Thursday, Friday, Saturday) for both assessment periods.	Tablet with a camera	Mild to moderate IDD adolescents (IQ 35–69), of sufficient cognitive ability to understand directions, and able to communicate through spoken language determined by an interview	Total Daily Energy Expenditure; Food Image-Assisted Record	Participants successfully completed their 3-day food records and self-collected DLW urine samples for 100% of the required days. Images were captured for 67.4 ± 30.1% of all meals recorded at assessment 1 and 72.3 ± 29.5% at assessment 2. The energy intake measured by IAR demonstrated acceptable test–retest reliability (ICC = 0.70). On average, IAR underestimated total energy intake by −299 ± 633 kcal/day (MPE = −9.6 ± 22.2%), however, there was a large amount of individual variability in differences between the IAR and TDEEDLW (range = −1703 to 430).
Wingo et al. (31)	6–17	65	Both	RCT	Healthcare center	Primary prevention	The study aimed to test the usability and preliminary efficacy of an e-health and telecoaching intervention that combines digital health resources and human interaction to achieve diet and physical activity behavior change, compared to telecoaching alone.	The study was conducted for 2 years and 3 months. Both groups received weekly phone calls from the telecoach for the first 6 weeks of the study, and every other week during the other 6 weeks. In addition, the e-TH group received access to the online platform POWERS.	Web-based telecoaching system Telephone	Age, lower extremity mobility disability, the ability to use hands and arms for exercise	Height and weight, dietary patterns, and physical activity.	There was no significant difference in coaching calls completed between groups. There were no significant differences in change in weight, fruit or vegetable intake, or physical activity levels.
Kawabata et al. (37)	13–16	171	Both	RCT	School	Primary prevention	The study aimed to investigate whether a school-based intervention targeting salient physical activity benefits and barriers grounded on the theory of planned behavior would promote young people’s participation in moderate-to-vigorous physical activity during leisure time and reduce the BMI of overweight students.	The students were initially tested in the control condition over 12 weeks (without delivering persuasive messages during the Physical Activity lessons). After they were tested during the intervention condition over 11 weeks (through delivering persuasive messages during the PA lessons)	Accelerometer	Age	BMI, International Physical Activity Questionnaire, Leisure-Time Physical Activity Participation Questionnaire, PA level via Accelerometer and psychological variables.	Results not available
Lubans et al. (38)	13–15	640	Both	Cluster RCT	School	Primary prevention	The study aims to assess the effectiveness of two physical activity promotion programs (NEAT and ATLAS) that have been modified for scalability and evaluate the dissemination of these programs throughout government-funded secondary schools	The study is conducted in two phases. In the first phase (cluster randomized controlled trial), 16 schools will be randomly allocated to the intervention or a usual care control condition. In the second phase, the Reach, Effectiveness, Adoption, Implementation and Maintenance (Re-AIM) framework will be used to guide the design and evaluation of program dissemination throughout New South Wales (NSW), Australia.	Mobile App	Attending 9-year classes	Muscular fitness, BMI, cardiorespiratory fitness, flexibility, resistance training skill competency, physical activity, self-reported recreational screen-time, sleep, sugar-sweetened beverage and junk food snack consumption, self-esteem and well-being	Results not available
Luque et al. (27)	8–13	334	Both	RCT	Health center	Secondary prevention	The study aims to evaluate the efficacy of a multicomponent motivational program for the treatment of childhood obesity, coordinated between primary care and hospital specialized services, compared to the usual intervention performed in primary care.	Every child in both the control and intervention group was visited monthly for 12 months, with the detection of anthropometric measures and received dietary recommendations and increased physical activity. The intervention group followed a motivational interview and could participate in a workshop on three different topics: Increasing physical activity (monitored with Fitbit) Food choices and balance, using healthy cooking methods.	Mobile App Wrist physical activity monitor	BMI > 97^th^ percentile	Body Composition, Vascular Function, Respiratory Function, Adherence to the Intervention	Results not available
Puigdomenech et al. (39)	13–16	525	Both	Quasi-experimental controlled cluster trial	School	Primary prevention		Participants are provided with a smartphone with the PEGASO apps installed for the duration of the study. At the 4th month of the study were introduced physical activity monitors (smart bracelet and smart sensor)	Activity bracelet T-shirt/cropped top with embedded textile electrodes Game Mobile App	Age	Anthropometric measurements, body composition, dietary habits, physical activity, sedentary behavior, and sleeping habits, knowledge/awareness about these health habits and user experience of the platform.	Results not available
Van Lippevelde et al. (32)	14–16	1,437	Both	Observational study	School	Primary prevention	The study aims to measure the effectiveness of an mHealth intervention named the ‘Snack Track School’ to improve adolescents’ snacking behavior	The adolescents in the intervention schools (three schools) will receive a four-week mHealth intervention while the control schools (the other three schools) will continue their usual practices. Participants earn credits on the nutritional value of the snack they consume; the healthier the snacks they consume, the more points they will receive. No negative points will be provided for unhealthy snacks.	Mobile App	Age	Healthy Snacking Index (FFQ), anthropometric measurements, cognitive variables (awareness, attitude, self-efficacy, habits, knowledge of the healthiness of snacks)	Results not available
Fleischman et al. (40)	10–17	40	Both	RCT	Health center	Primary prevention	The study aims to compare BMI changes between two arms: primary care providers (PCPs) in-person clinic visits plus obesity specialist tele-visits (group 1) and PCP in-person clinic visits only (group 2), with ongoing tele-consultation between PCPs and obesity specialists for both arms.	Using a cross-over protocol, Group 1 had PCP visits + Specialist tele-visits during the first 6 months and PCP visits only during the second 6 months, and Group 2 followed the opposite sequence.	Tele-consultation and tele-visits system Webcams or secure iPads Messaging and social media	BMI ≥ 95th percentile	Anthropometry, Blood Pressure, Diet and Physical Activity	BMI decreased more for Group 1 vs. Group 2 at 3 months following frequent tele-visits. At 6 months (primary outcome), BMI was lower than baseline within Group 1 but not Group 2. However, the decrease in BMI at 6 months did not differ between groups. After crossover, BMI remained lower than baseline for Group 1 and dropped below baseline for Group 2.
Zhang et al. (41)	16–18	46	Both	Pre–post-pilot feasibility randomized trial	School	Primary prevention	The study aims to assess the feasibility and preliminary effects of an mHealth nutrition education and mindful snacking intervention for weight loss and improved dietary practices among adolescents with overweight	Nutrition education to promote adolescents’ healthy food choices for snacking; mindfulness-based healthy eating skills to reduce snacking in the absence of hunger, and planning to improve the usage of mindful snacking skills.	Smartphone Video messages	BMI ≥ 24 kg/m2	BMI; Dutch Eating Behavior Questionnaire (DEBQ); Food Cravings Questionnaire—Trait-reduced (FCQ-T-r); Weight Efficacy Lifestyle Questionnaire Short Form (WEL-SF); Beverage and Snack Questionnaire (BSQ); Acceptability	The main effect of time on all outcome measures; participants reported positive experiences and high levels of satisfaction with taking part in the intervention.
Chagas et al. (33)	13–16	319	Both	RCT	School	Primary prevention	The study aims to assess the impact of a game-based nutritional intervention on food consumption, nutritional knowledge and self-efficacy in the adoption of healthy eating practices	Intervention group participants were instructed to play a digital game developed for the study, on their own, for 7–17 days. The control group was not provided with any game or material during the study.	Gaming	Age	Age, sex, monthly family income, maternal education level, dietary perceptions and practices, nutritional knowledge and self-efficacy in the adoption of healthy eating practices	Significant reductions in the intervention group compared with the control group for the following variables: habit of eating while watching TV or studying, and having meals at fast food restaurants. Increased knowledge of the effects of fruit and vegetable consumption as well as improved self-efficacy in the adoption of healthy eating practices
Dzielska et al. (22)	15	1,111	Fem.	RCT	School	Primary prevention	The study aims to evaluate the effectiveness of a mHealth program on changes in healthy eating and physical activity among overweight and non-overweight female students.	The intervention included behavioral and environmental components. Self-efficacy was modeled by setting goals, observing others, and receiving feedback from technologies (fitness band, apps) that supported self-monitoring.	Mobile app Fitness Band Social (Facebook)	Age, Sex	Health Behavior Indicators, Physical activity, Self-Efficacy—Personal Competence Scale, Body Weight	A significant effect of self-efficacy with the interaction of body weight status on improvement in eating behavior and physical activity among adolescent girls.
Folkvord and de Bruijne (34)	13–16	132	Both	RCT	School	Primary prevention	The study aims to test if promoting red peppers on social media through a popular social influencer increases subsequent actual vegetable intake among adolescents	Adolescents answered a questionnaire divided into two parts. After answering the first part of the questionnaire, adolescents were exposed to the Instagram post and then answered the second part.	Social Media (Instagram)	Age	Vegetable consumption, Parasocial interaction, and Persuasion knowledge	No significant interaction effect of type of Instagram post and parasocial interaction on vegetable intake was observed. Next, no significant interaction effect of type of Instagram post and persuasion knowledge on vegetable intake was observed.
Sousa et al. (35)	12–16	361	Both	Non-RCT	School	Primary prevention	To assess the effectiveness of an mHealth intervention on lifestyle change to promote healthy behaviors in adolescence	The Intervention with Mobile app included educational resources, self-monitoring features, interactive training modules and motivational tools. The intervention was evaluated at baseline (T0) and post-intervention (6 months: T1).	Mobile app, Gaming	Age	Adolescent lifestyle profile (ALP), Body image dissatisfaction, eHealth literacy	The mHealth intervention is more effective than the standard school-based intervention in the promotion of healthy behaviors. A small effect of the mHealth intervention on nutrition, positive life perspective, and global lifestyle has been found.

### 3.3 Quality assessment

The study quality assessment is reported in Table 4.

**Table 4:** Results of the study quality assessment.

General information on the study	Selection bias	Design	Confunders	Blinding	Data collection methods	Withdrawals and dropouts	Final quality assessment
Stasinaki et al. (24)	Strong	Strong	Moderate	Weak	Strong	Strong	Strong
Vidmar et al. (25)	Strong	Moderate	Strong	Weak	Strong	Strong	Strong
Verswijveren et al. (28)	Strong	Strong	Weak	Moderate	Strong	Strong	Strong
Sousa et al. (36)	Strong	Moderate	Moderate	Moderate	Moderate	Weak	Moderate
Hagman et al. (26)	Strong	Moderate	Strong	Moderate	Strong	Moderate	Moderate
Ridgers et al. (23)	Strong	Strong	Moderate	Moderate	Moderate	Strong	Moderate
Salahshornezhad et al. (29)	Strong	Strong	Moderate	Moderate	Strong	Moderate	Moderate
Ptomey et al. (30)	Strong	Moderate	Moderate	Moderate	Moderate	Moderate	Moderate
Wingo et al. (31)	Strong	Moderate	Moderate	Weak	Strong	Strong	Moderate
Kawabata et al. (37)	Strong	Moderate	Strong	Moderate	Strong	Weak	Moderate
Lubans et al. (38)	Strong	Strong	Moderate	Moderate	Strong	Weak	Moderate
Luque et al. (27)	Strong	Moderate	Strong	Strong	Strong	Weak	Strong
Puigdomenech et al. (39)	Strong	Moderate	Strong	Moderate	Moderate	Weak	Moderate
Van Lippevelde et al. (32)	Strong	Moderate	Strong	Weak	Strong	Weak	Weak
Fleischman et al. (40)	Strong	Strong	Strong	Strong	Strong	Moderate	Strong
Zhang et al. (41)	Strong	Moderate	Moderate	Weak	Strong	Weak	Weak
Chagas et al. (33)	Moderate	Strong	Moderate	Strong	Moderate	Weak	Moderate
Dzielska et al. (22)	Weak	Weak	Weak	Weak	Moderate	Moderate	Weak
Folkvord and de Bruijne (34)	Strong	Moderate	Weak	Strong	Weak	Strong	Weak
Sousa et al. (35)	Moderate	Moderate	Strong	Strong	Strong	Strong	Strong

## 4 Discussion

This work systematically reviewed obesity prevention interventions in children and adolescents supported by digital solutions. Twenty studies with various experimental designs, characteristics, durations, and intensities were identified after conducting a deep search across two databases. The designs of the selected studies were rigorous: *n* = 10 studies were Randomized Controlled Trials (RCT); *n* = 2 were cluster-RCT; *n* = 2 were non-RCT; *n* = 3 were observational; *n* = 1 was a Pragmatical clinical Trial; *n* = 1 was a quasi-experimental controlled cluster trial; and *n* = 1 was a pre–post feasibility randomized trial. The studies described interventions in a population ranging from the age of 4–18 years. *N* = 18 studies enrolled female and male populations, *n* = 2 studies enrolled only girls (22, 23). The studies described interventions for weight control to prevent obesity as effective in 50% of the cases (*n* = 10), in 20% of the cases, there was no significant increase in outcomes and in 30% (*n* = 6) of the studies, the results were unavailable. According to the literature (13, 14), the results of the present review suggest that technology can support multidimensional interventions for obesity prevention, often combining and integrating different digital solutions. Most of the studies (*n* = 8; 40%) included nutritional intervention, *n* = 3 (15%) studies included a physical activity intervention, *n* = 4 studies (19%) included both interventions, *n* = 5 (23%) studies included, in addition to physical activity and nutritional intervention, psychological or behavioral interventions. The review aims to provide a perception of the state-of-the-art digital interventions to prevent obesity in adolescents. Heterogeneity among studies represents the most critical limitation of this review, which affected the generalizability of the results and the comparison between studies. This did not allow for a meta-analysis because the study design, age, objectives, assessment measures and tools are different and do not allow for an aggregate analysis. Despite this, the review confirmed that interventions for preventing obesity in adolescents mainly concerned adherence to physical activity and a greater awareness of healthy nutritional prescriptions. Mobile apps are the most effective digital tools to promote changes in adolescents’ behaviors. From a clinical point of view, there is an increasing demand for tools to monitor adherence to nutritional prescriptions and physical activity, such as activity trackers and innovative diet diaries. Some of the studies are directly focused on interventions that impact clinical parameters such as BMI, weight and blood pressure (24–27) while some focus on behavior change for nutrition and physical activity (23, 28–35), and others on both (22, 36–41). The studies implemented at healthcare centers produced significant improvements in clinical parameters, demonstrating their greater interest in the clinical component of the intervention (24–26, 40). The studies on lifestyle changes that produced better results are the ones developed at school (22, 33, 35, 36, 41). This demonstrates the importance of the school setting for behavior change in the target population. Only one study produced significant results on adherence to monitoring eating habits and clinical parameters in healthcare setting (30). Although some studies do not describe statistically significant improvements, they encourage further studies with larger samples and include cost-effectiveness analysis (36). A key component of Verswijveren et al., intervention is the activity tracker that self-monitor physical activity, increasing the adolescents’ awareness of low activity levels, thus perceiving more barriers than those in the control group (28) According to literature, some level of intrinsic motivation must be present within the user to ensure continued use of digital solutions for lifestyle changes (42). Ridgers et al. (23), in describing the disappointing results in improving students’ adherence to physical activity, highlighted the need to combine wearable activity trackers with appropriately engaging digital resources. Wingo et al. weight control intervention failed because it was necessary to balance the need for greater child involvement with technology, and the parents’ desire for less interaction (31). Folkvord and de Bruijne argue that the failure of social media awareness campaigns depends on the careful development of content, rather than on the ability of influencers to impact the habits of teenagers (34).

### 4.1 Digital tools

Most studies (*n* = 11; 55%) use mobile apps to implement the intervention but are associated with other solutions (*n* = 7). The apps described in the various studies have different objectives and act on several dimensions (i.e., physical, nutritional, behavioral, etc.) with different modalities. Five studies implemented a digital intervention based on gaming. In the study conducted by Sousa et al. (35), a game-based learning process was developed through a mobile app. The tool includes prizes, points, and progress to the adolescent reported to be in the wall of fame. In another study (39), in addition to the reward system and the gamification module that allows users to get one-time awards for the selected target behavior, an avatar was developed on a mobile app that educates, cares and empowers adolescents in developing healthy habits. Through the avatar, the tool provides the user with educational and motivational messages and updates on their results and the results shared by their friends. Also, in the study conducted by Salahshornezhad et al. (29), there is a game-based intervention focusing on nutritional education accessible via smartphone. The mobile app enables aerobic exercise under the supervision of a trainer, along with cognitive behavioral therapy sessions specifically designed for adolescents by a clinical psychologist. The “Snack Track School” app (32) reproduces a virtual school environment with classrooms, gym, bicycle area, bathrooms, etc., where users have their locker, their own Snack Track tool and their own “anonymous” avatar, which they can customize. During the four-week intervention, users receive a storyline with challenges that help them track their eating habits. The “Snack Track School” tool allows users to earn credits/points based on the nutritional value of their snacks, performing positive reinforcement to influence automatic behavior. Rango Cards is a game-based mobile app that utilizes virtual reality (33). Rango Cards occurs in a virtual school where players visit different environments, including the canteen. The game is divided into phases that address food classification, healthy eating practices, the importance of cooking, misleading advertising, and reading food labels to understand their nutritional content. Other apps used in the study were integrated with the wearable activity tracker to monitor physical exercise. The app’s interface was often attractive to encourage its use by adolescents and make understandable the progress achieved in the different sessions (22, 23, 26–28, 39). The use of smartphones for nutritional interventions based on motivational reinforcement, diet control, and support systems in patients with intellectual disabilities is interesting. In these two studies, parents were also involved when necessary (30, 31).

### 4.2 Setting

The settings for enrollment of adolescents that received interventions for the prevention of obesity were schools (*n* = 13; 65%), healthcare centers (*n* = 6; 30%), such as pediatric services, and patients’ homes (*n* = 1; 5%). School is the most diffused place for implementing interventions to prevent obesity, probably because it provides a practical setting for educating about the consumption of healthy foods. Schools represent an optimal learning environment that reaches children from all socio-economic backgrounds (43). There is a need to prove the effectiveness of interventions in schools by implementing a multi-component educational approach dedicated to the children directly; the school environment, including school staff; and family members. This multi-component approach was often not described in the studies examined, except for patients with disabilities. A limitation of this review is the lack of a more complete understanding of the contextual factors that could influence behavior change (44). No information was found about public campaigns to raise awareness of adopting healthier behaviors and, therefore, motivate the use of digital tools. There is a lack of information about the presence of school policies that discourage the intake of sugar-sweetened beverages. Without this information, the evaluation of the success of digital interventions remains partially explained. However, further investigation is needed in this area to obtain more data. The studies implemented in the healthcare facilities enrolled children who already had a condition of overweight or obesity (≥ 85th percentile BMI). In these cases, interventions mediated by digital technologies allow monitoring patients’ behaviors in their living environment, representing a sustainable alternative to the outpatient visit for weight management. In the two studies in which the child was recruited at home, the children had intellectual disabilities. Adolescents with physical disabilities are at greater risk because physical inactivity, poor food intake and unhealthy lifestyle behaviors are neglected compared to the clinical and social management of disability. They are also more reluctant to participate in health promotion activities at school or in the community, and are much more likely to be sedentary and eat poorly. In this scenario, digital solutions to prevent obesity represent a valid and very effective strategy to improve adherence to healthy lifestyles and motivate the patient to socialize.

### 4.3 Level of intervention

Distinguishing between primary, secondary, or tertiary prevention is essential to identify the factors that drive weight gain among healthy adolescents and the achievement of therapeutic targets in overweight or obese adolescents (45). Primary prevention interventions aim to maintain a healthy weight, estimated at a BMI between the 5th and 85th percentiles in children and adolescents, corresponding to an adult BMI of 18.5–25 kg/m^2^. The present review revealed that most primary prevention interventions were conducted in schools (*n* = 13; 65%). *n* = 8 studies demonstrated that digital solutions are effective in supporting obesity prevention interventions. A meta-analysis study suggested that obesity prevention interventions for children aged 6–18 years at school have a small beneficial impact on BMI (46). The great diversity of interventions suggests that a more comprehensive assessment is required to identify the clinical and behavioral factors that allow effective interventions and to inform future obesity prevention public health policy (32).

Secondary prevention interventions aim to control weight in overweight individuals, thus keeping the BMI stable or reducing it from the overweight level to a healthy weight. The studies examined in the present review that provided a secondary prevention intervention were *n* = 4 (20%). Two studies enrolled adolescents in a healthcare center (25, 27); in *n* = 1 study, the adolescents were enrolled by a healthcare service integrated with school (26); and in *n* = 1 they have been enrolled at school (26). Except one, all studies confirmed the effectiveness of the interventions. Vidmar et al. compared a traditional clinical intervention versus an intervention supported by digital technology for weight loss in adolescents (25). Results demonstrated high retention and adherence rates, and reduced zBMI and %BMIp95 are more cost-effective than in the traditional clinical intervention. Hagman E. et al., 2022 confirmed that a digital support system for personalized weight-loss target curve and daily weight measurements, shared with family and healthcare professionals (HCP) is more effective than a standard childhood obesity treatment (26).

Tertiary prevention aims to hinder severe obesity (i.e., BMI > 95th percentile, corresponding to adult BMI > 30 kg/m^2^), prevent weight regain, and avoid comorbidities in children and adolescents who are living with obesity. In the present review, only one study implemented a tertiary prevention intervention. The intervention consists of counseling via a mobile app to support overweight or obese adolescents in adopting a healthy lifestyle, compared to a multi-component behavior change program. The study showed a significant decrease in BMI-SDS in the control group, rather than in the mobile app group. Muscle mass, strength and agility improved significantly in both groups. The group that used the mobile app significantly reduced body fat. The average daily usage of mobile apps is very encouraging (24). For this reason, the author suggested digital interventions to manage or sustainably follow up adolescent obesity, becoming an essential part of teletherapy and offering additional services to patients and families.

## 5 Conclusion

The review aimed to identify studies that implemented interventions supported by digital technologies to prevent obesity or promote weight control. The prevention levels, the setting, the digital tool adopted, and the effectiveness of the interventions were identified in the selected studies. The interventions mainly concerned nutritional aspects and physical activity. The studies demonstrated that motivational and psychological support play a fundamental role in the success of the intervention. The most widespread solutions were mobile apps and wearable devices for monitoring physical activity, often integrated. Interventions spread via social media are also popular. The use of smartphones is strategic. This review confirms that IT-supported personalized secondary prevention interventions for the treatment of obesity, with a strong integration between the different actors (i.e., health service, school, parents), present positive results in terms of weight loss. It is necessary to encourage integration among the various actors interacting with adolescents in multiple settings (School, Healthcare center, Home, Leisure-time facility). The widespread use of digital devices ensures new possibilities both for prevention strategies and personalized treatments to curb the expansion of the obesity epidemic (47). Investing in new digital technologies to prevent obesity is advantageous in terms of the effectiveness and sustainability of interventions, with positive impacton service delivery organization. This work aims to contribute to the discussion on designing and implementing an innovative diagnostic and therapeutic path. The present review suggests that the efficacy of digital solutions remains limited; they can play a strategic role in the interventions, especially if they provide different integrated solutions, connected to traditional clinical interventions. Mobile applications, web-based tools, text messages, portable monitoring devices/personal digital assistants (PDAs) and pedometers allow accessible interaction, frequent contact, and data monitoring through mobile applications, which improve short-term outcomes (48).

## Data Availability

Publicly available datasets were analyzed in this study. This data can be found at: Medical Literature Analysis and Retrieval System Online (MEDLINE) and Excerpta Medica Database (EMBASE).
